# Another way to skin a c(o)a(rc)t

**DOI:** 10.1093/icvts/ivac141

**Published:** 2022-05-30

**Authors:** Karthik V Ramakrishnan

**Affiliations:** Division of Pediatric Cardiovascular Surgery, LeBonheur Children’s Hospital and University of Tennessee Health Sciences Center, Memphis, TN, USA

**Keywords:** Aortic arch, Thoraco-sternotomy, Aneurysm

In this issue of the journal, Koizumi and colleagues [1] have demonstrated the use of an L-shaped thoraco-sternotomy incision to perform resection and repair of a distal arch aneurysm with pseudo-coarctation in a 16-year old. This approach has been described extensively described for total aortic arch replacement in adults [2–4], and the authors have adapted this approach to successfully treat their patient. The authors must be congratulated on achieving an immensely satisfactory outcome with some out-of-the box thinking in a complex case.

There is no doubt that the L-shaped incision provides excellent exposure for total arch replacement [3]. This incision combines the advantages of a median sternotomy as well as a thoracotomy. The extension makes the distal end of the anastomoses more easily accessible as compared to a sternotomy and facilitates the use of total body perfusion during the operation as the authors have demonstrated. Notwithstanding the unresolved debate regarding deep hypothermic circulatory arrest versus selective cerebral perfusion, the L-approach makes the operation easier than either a sternotomy or a thoracotomy alone irrespective of the bypass technique used. The cosmetic appearance is a small price to pay for achieving an excellent surgical outcome. In the paediatric world, I would probably think of using this technique in cases of complex redo coarctations.

## References

[1] Koizumi J , Takuya G, Saiki H, Kin H. Distal arch replacement for aortic aneurysm associated with pseudocoarctation through the L-incision approach. Interact CardioVasc Thorac Surg 2022. https://doi.org/10.1093/icvts/ivac094. 10.1093/icvts/ivac094 PMC9336573 35417001

[2] Abe T , Suenaga H, Oshima H, Araki Y, Mutsuga M, Fujimoto K et al An L-shaped incision for an extensive thoracic aortic aneurysm and coronary artery bypass using the left internal thoracic artery. Aorta (Stamford) 2015;3:86–9. 26798763 10.12945/j.aorta.2015.14-061 PMC4686355

[3] Oishi Y , Sonoda H, Tanoue Y, Nishida T, Tokunaga S, Nakashima A et al Advantages of the L-incision approach comprising a combination of left anterior thoracotomy and upper half-median sternotomy for aortic arch aneurysms. Interact CardioVasc Thorac Surg 2011;13:280–3. 21680550 10.1510/icvts.2011.266791

[4] Tokuda Y , Oshima H, Narita Y, Abe T, Mutsuga M, Fujimoto K et al Extended total arch replacement via the L-incision approach: single-stage repair for extensive aneurysms of the aortic arch. Interact CardioVasc Thorac Surg 2016;22:750–5. 26932664 10.1093/icvts/ivw034 PMC4986779

